# Clinical characteristics of anti-*N*-methyl-d-aspartate receptor encephalitis in patients with a long-term history of mental disorders

**DOI:** 10.1186/s40001-022-00664-5

**Published:** 2022-03-10

**Authors:** Hai-Yang Wang, Xiao-Yu Yang, Jinming Han, Huakun Liu, Zhong-Rui Yan, Zhanhua Liang

**Affiliations:** 1 Department of Neurology, The First Affiliated Hospital of Dalian Medical University, No. 222, Zhongshan Road, Dalian, 116011 Liaoning China; 2 Department of Neurology, Jining No. 1 People’s Hospital, Jining, 272000 China; 3 Department of Psychiatry, School of Mental Health, Jining Medical University, Jining, 272000 China; 4 Department of Neurology, Xuanwu Hospital, Capital Medical University, Beijing, 100053 China

**Keywords:** Anti-*N*-methyl-d-aspartate receptor encephalitis, Mental disorders, Immunotherapy

## Abstract

**Background:**

Anti-*N*-methyl-d-aspartate (NMDA) receptor encephalitis is an autoimmune disorder characterized by complex neuropsychiatric syndromes during disease onset. Although this disease has been well documented in the last decade, clinical characteristics of anti-NMDA receptor encephalitis in patients with long-term diagnostic history of mental disorders remain unclear.

**Methods:**

Here, we reviewed and analyzed series of anti-NMDA receptor encephalitis patients with a long-term medical history of psychiatric disorders through a review of literature using PubMed, web of science and Embase database. In addition, we described a patient of anti-NMDA receptor encephalitis with a long-term history of major depressive disorder.

**Results:**

A total of 14 patients with anti-NMDA receptor encephalitis and a long-term history of mental disorders were included in our study. We found that most patients were adult (92.9%) and female (78.6%). These patients often first visited a psychiatric department (71.43%). The mean disease course of psychiatric disorders was more than 9 years. Speech impairment (71.4%), abnormal behaviors (64.3%), and catatonia (64.3%) were the most common clinical symptoms. Most patients (85.7%) had a satisfactory prognosis after immunotherapy.

**Conclusion:**

Anti-NMDA receptor encephalitis in individuals with mental disorders is an underestimated condition, yet it presents complex clinical symptoms. Mental and behavioral impairments are more frequently observed in newly diagnosed anti-NMDA receptor encephalitis patients with a long-term history of mental disorders than those without mental illness. A diagnosis of anti-NMDA receptor encephalitis should be considered when patients with mental illness show sudden fluctuations in psychiatric symptoms.

## Introduction

Anti-*N*-methyl-d-aspartate (NMDA) receptor encephalitis is an autoimmune disorder of the central nervous system mediated by anti-NMDA receptor antibodies [1]. The major symptoms of anti-NMDA receptor encephalitis are complex neuropsychiatric symptoms including delusions, hallucinations, psychomotor agitation, altered consciousness, aggressiveness, speech impairment, movement disorders, seizures, cognitive deficits, and autonomic instability [2]. Anti-NMDA receptor encephalitis can often be misdiagnosed as other diseases, such as viral encephalitis, psychiatric disorder, and status epilepticus [2]. Despite the presence of neurological comorbidities, many patients were first evaluated by a psychiatrist due to obvious psychiatric symptoms [1, 3].

Emerging evidence suggests that rapid new onset of psychiatric symptoms in a person without a medical history of mental illness could serve as an important alarm to suspect anti-NMDA receptor encephalitis [1, 4, 5]. However, clinical characteristics of newly diagnosed anti-NMDA receptor encephalitis patients with a long-term history of psychiatric disorders remain unclear. Herein, we reviewed and analyzed a series of anti-NMDA receptor encephalitis patients with a long-term medical history of psychiatric disorders through a review of the literature. In addition, we described a patient with anti-NMDA receptor encephalitis and a long-term history of major depressive disorder. Therefore, in the present study, we aimed to describe the clinical features and establish a clinical position of anti-NMDA receptor encephalitis.

## Methods

### Representative case presentation

A 28-year-old male was transferred to the department of neurology from a psychiatric hospital, suffering from confusion, agitation, speech impairment, rigidity, and fever. He was diagnosed with major depressive disorder 12 years ago with symptoms of depressive mood, desperation, anxiety, insomnia, fear, upset, slurred speech, and decreased speech output. The patient’s initial onset of depression was subacute in appearance with fluctuating episodes. However, he failed to take medications regularly. Fourteen days before admission, the patient experienced confusion, catatonia, and agitation. Four days prior to admission, he displayed speech impairment, anorexia, and marked rigidity of the torso. He then visited a local psychiatric hospital and was given a sedative treatment (diazepam, 20 mg daily). The patient developed a fever (2 days before admission), with a temperature of up to 38 °C. The patient’s guardian reported that the patient was not taking antipsychotics in the 3 months prior to the onset of illness. On admission, his body temperature reached a maximum of 40 °C. Neurological examinations revealed lethargy, speech impairment, and rigidity of the neck and torso.

Laboratory tests showed several abnormalities: elevated blood white cells (1.55 × 10^10^/L, normal range 3.5–9.5 10^9^/L) and C-reactive protein (23.3 mg/L, normal range 0–10 mg/L), and decreased free triiodothyronine (3.36 pmol/L, normal range 3.8–7.0 pmol/L), total thyroxine (61.50 nmol/L, normal range 69.97–152.52 nmol/L), and thyroid stimulating hormone (0.28 μIU/mL, normal range 0.34–5.6 μIU/mL). Anti-thyroid peroxidase (anti-TPO) and anti-thyroglobulin (anti-TG) antibodies were negative. The lumbar puncture showed that the CSF pressure was 160 mmH_2_O. CSF biochemistry indicated that white blood cell count was 4 × 10^6^/L (normal range 0–8 × 10^6^/L), total cell counts was 4 × 10^6^/L, glucose was 3.9 mmol/L (normal range 2.8–4.5 mmol/L), protein was 0.36 g/L (normal range 0.15–0.45 g/L), and chloride was 132 mmol/L (normal range 111–123 mmol/L). Autoimmune encephalitis antibodies in the CSF and serum were assessed by an indirect immunofluorescence assay (rat hippocampal tissues) and a cell-based assay employing HEK cells transfected with the respective antigens. The CSF was positive for anti-NMDA receptor antibodies (IgG, 1:1), while the serum was negative. Tests were repeated using new samples and other equipment to confirm the above positive finding. Other autoantibodies including AMPA1-IgG, AMPA2-IgG, LGI1-IgG, CASPR2-IgG, and GABABR-IgG were negative in both CSF and serum. Paraneoplastic neuronal antibodies including anti-Hu, anti-Ri, anti-Yo, anti-Ma2, anti-Tr, anti-ANNA-3, anti-PCA-2, anti-GAD, anti-amphiphysin, anti-CV2, anti-SOX1, and anti-Ma2/Ta were unremarkable in the CSF and serum. Cranial computed tomography scans were normal.

A diagnosis of anti-NMDA receptor encephalitis was considered; the patient received intravenous immunoglobulin (25 g daily for 5 days) and high-dose intravenous methylprednisolone (250 mg daily for 5 days). Midazolam (0.5 mg/kg per hour, intravenous) was used for sedation. However, the patient’s symptoms worsened and he subsequently developed seizures, tachycardia, and central hypoventilation. The patient then developed a severe pneumonia, abdominal cavity infection, and sepsis. He continued to progressively deteriorate and died due to respiratory and circulatory failure 14 days after admission.

### Literature search and selection

To understand further the clinical characteristics of anti-NMDA receptor encephalitis in patients with a coexisting medical history of mental disorders, we performed an extensive literature search to identify other cases (published between September 2007 and October 2020). We searched PubMed, Web of Science, and Embase using the following terms: “anti-NMDA receptor encephalitis”, “anti-*N*-methyl-d-aspartate receptor encephalitis”, “psychiatric disorder”, “psychosis”, “psychoses”, “mental disorder”, and “mental disturbance.” Long-term mental disorders were defined as a disease duration > 1 year [6]. Non-English language articles were excluded from our study. An improvement in the outcome was defined as an improvement in the patient’s ability to perform activities of daily living, including mental, cognitive, and physical abilities, compared with the onset of the illness. The symptoms of “speech impairment” were defined as incoherent speech, speaking cessation, verbally unresponsive, limited verbal output, and slurred speech.

## Results

### Clinical characteristics

**Fig. 1:**
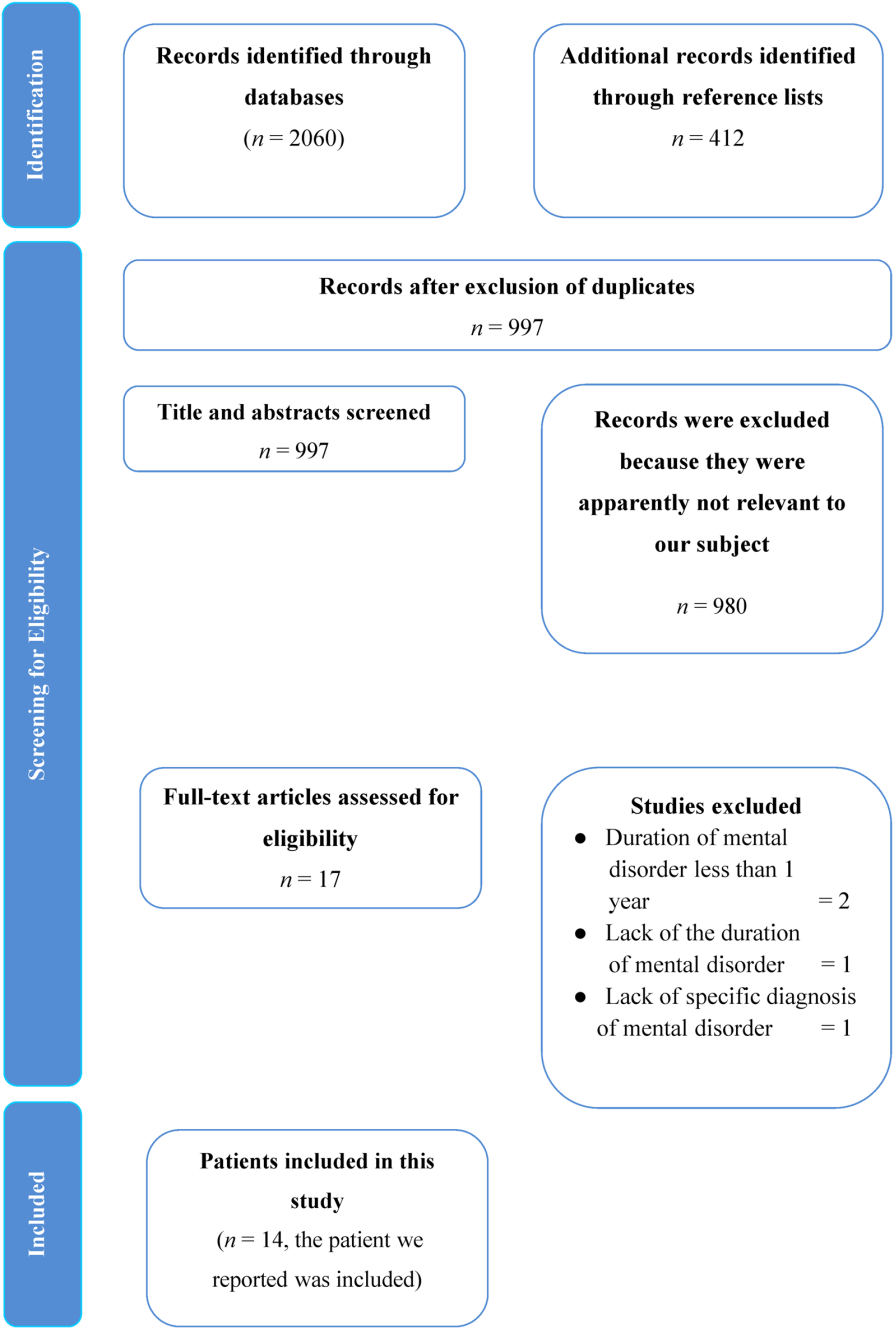
Flow diagram of processes of article selection

A total of 2472 articles were identified upon the initial search. After removing duplicates, 997 records were available for scrutiny. After screening the titles and abstracts of these articles, 980 apparently irrelevant articles were excluded. Two articles were excluded due to a short-term duration of mental disorders. Another two articles were excluded because they lacked information of disease duration and specific mental diagnosis. Together with our own report, 14 cases were identified (Fig. 1).

**Fig. 2:**
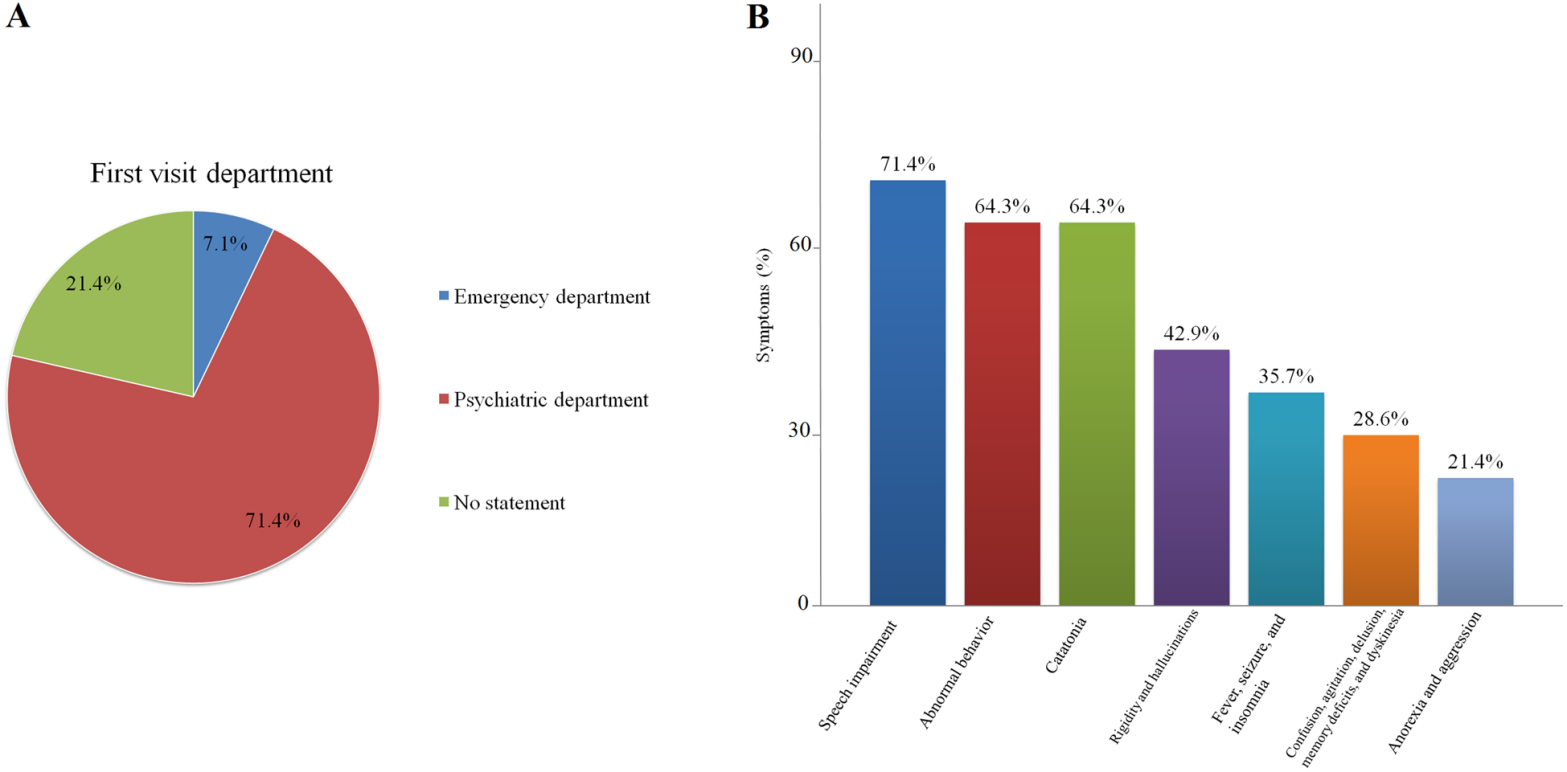
Distribution of the first visit of department (**A**) and clinical symptoms (**B**) in newly diagnosed anti-NMDA receptor encephalitis patients with a long-term history of mental disorders. N = 14

The clinical characteristics of these 14 cases are summarized in Tables 1 and 2. Specifically, the age of disease onset varied between 16 and 52 years of age, with an average of 35 years. Thirteen patients (92.9%) were adults and one was a teenager. Eleven patients were female (78.6%). Nine patients (71.43%) first visited the psychiatric department, one patient first visited the emergency department, while three patients (21.43%) did not report the above information clearly (Fig. 2A). Among 14 patients, four of them suffered from depressive disorders (three of them were diagnosed with major depressive disorder), three patients suffered from bipolar disorder, three patients had schizophrenia, two patients had schizoaffective disorder, one patient had attention deficit hyperactivity disorder, and one patient had autism spectrum disorder. The duration of psychiatric disorders varied between 1 and 22 years, with an average of 9.8 years. Thirteen patients (92.9%) showed positive anti-NMDA receptor antibodies in the CSF. Five patients (35.7%) showed positive anti-NMDA receptor antibodies in both CSF and serum. Only one case was positive in the serum alone. Two of them (14.3%) reported malignancies (cervical cancer and ovarian teratoma). The patient with cervical cancer was positive for NMDA receptor IgG in CSF and negative in serum, and patient with ovarian teratoma was positive for NMDA receptor IgG in SCF and serum. As shown in Fig. 2B, ten patients with psychiatric disorders (71.4%) experienced speech impairment during the duration of anti-NMDA receptor encephalitis. Furthermore, nine cases (64.3%) had abnormal behaviors. Catatonia was observed in nine cases (64.3%). Rigidity and hallucinations were reported in six patients (42.9%). Fever, seizures, and insomnia occurred in five patients (35.7%). Confusion, agitation, delusion, memory deficits, and dyskinesia were found in four patients (28.6%). Three patients (21.4%) presented with anorexia and aggression. A total of 13 patients underwent brain magnetic resonance imaging (MRI) scans. Nine patients (69.2%) did not show significant abnormalities, two patients (15.4%) had several small ischemic foci in the frontal lobe, one patient had dural thickening and meningeal contrast enhancement, one patient had frontoparietal cortical atrophy.

**Table 1:** Overview of clinical features of newly diagnosed anti-NMDA receptor encephalitis patients with a long-term history of mental disorders

No.	Age/sex	Initial visit	Psychiatric history (years)	Malignancies	CNS symptoms	NMDA receptor IgG	Neuroimaging	References
1	28/M	Psychiatry clinic	Major depressive disorder (12)	No	Fever, confusion, abnormal behaviors, catatonia, rigidity, seizures, speech impairment, agitation, tachycardia and anorexia	Serum: (−)	CT: no significant abnormality	Our case
						CSF: (1:1)		
2	52/F	Psychiatry clinic	Major depressive disorder (6)	Cervical cancer	Confusion, delusions, hallucinations, abnormal behaviors, speech impairment, and exacerbated depressed mood	Serum: ns	MRI: several small ischemic foci in frontal lobes	Rong et al. [7]
						CSF(1:100)		
3	52/M	Psychiatry clinic	Major depressive disorder (22)	No	Fever, seizures, insomnia, hallucinations, abnormal behaviors, poor cognition, speech impairment, tachycardia, hypopiesia and exacerbated depressed mood	Serum: (+)	MRI: no significant abnormality	Torgovnick et al. [8]
						CSF: (+)		
4	19/F	ns	Depressive disorders (1)	No	Headache, delusions, rigidity, insomnia, catatonia, papilledema, apathy and anorexia	Serum: ns	MRI: dural thickening and meningeal contrast enhancement	Caglayan et al. [9]
						CSF: (1:100)		
5	34/F	Psychiatry clinic	Bipolar disorder (10)	No	Fever, abnormal behaviors, speech impairment, catatonia, hallucinations, seizure, tachycardia, impaired memory, and orofacial dyskinesia	Serum: (+)	MRI: no significant abnormality	Simabukuro et al. [10]
						CSF: (+)		
6	52/F	Emergency	Bipolar disorder (8)	No	Speech impairment, rigidity, catatonia, urinary incontinence, apathy, ataxia, and dyskinesia	Serum: ns	MRI: frontoparietal cortical atrophy	Hanagasi et al. [11]
						CSF: (+)		
7	47/F	Psychiatry clinic	Bipolar disorder (4)	No	Aggression, catatonia, violence, anorexia, altered consciousness, stupor, and negativism	Serum: ns	MRI: no significant abnormality	Yoshimura et al. [12]
						CSF: (+)		
8	48/F	ns	Schizoaffective disorder (19)	No	Confusion, hallucinations, catatonia, abnormal behaviors, excitement, negativism, and stupor	Serum: ns	MRI: no significant abnormality	Yoshimura et al. [12]
						CSF: (+)		
9	38/F	Psychiatry clinic	Schizoaffective disorder (14)	No	Fever, confusion, speech impairment, hallucinations, seizures, insomnia, catatonia, memory impairment, agitation, and impaired attention	Serum: (+)	MRI: no significant abnormality	Heekin et al. [13]
						CSF: ns		
10	25/F	Psychiatry clinic	Schizophrenia (7)	No	Confusion, rigidity, insomnia, abnormal behaviors, speech impairment, delusions, dyskinesia, aggressiveness, and memory impairment	Serum: ns	MRI: a small ischemic focus in the frontal lobe	Huang et al. [14]
						CSF: (+)		
11	25/F	Psychiatry clinic	Schizophrenia (4)	No	Fever, tachycardia, abnormal behaviors, speech impairment, catatonia, agitation, rigidity, rhythmic orofacial grimacing, and autonomic instability	Serum: (1:640)	MRI: no significant abnormality	Conroy et al. [15]
						CSF: (1:160)		
12	33/M	Emergency	Schizophrenia (6)	No	Headache, rigidity, abnormal behaviors, speech impairment, catatonia, seizures, hallucinations, delusions, insomnia, psychomotor agitation, catatonic symptoms, and memory deficits	Serum: ns	MRI: no new changes compared with previous lesions	Ponte et al. [16]
						CSF: (+)		
13	16/F	Psychiatry clinic	Attention deficit hyperactivity disorder (4)	No	Confusion, abnormal behaviors, speech impairment, tachycardia, perceptual disturbances, and left facial droop	Serum: (1:10)	MRI: no significant abnormality	Fields et al. [17]
						CSF: (1:5)		
14	23/F	ns	Autism spectrum disorder (20)	Ovarian teratoma	Aggressiveness, dyskinesia and altered consciousness	Serum: (+)	MRI: no significant abnormality	Kurita et al. [18]
						CSF: (+)		

M, male; F, female; ns, no statement; CNS, central nervous system; NMDA, *N*-methyl-d-aspartate; CSF, cerebrospinal fluid; +, positive

**Table 2:** Immunotherapy, follow-up, and outcomes of newly diagnosed anti-NMDA receptor encephalitis patients with a long-term history of mental disorders

No.	Immunotherapy during hospitalization	Immunotherapy after discharge	Follow-up	Outcomes	References
1	First-line therapy	None	None	Died	Our case
	Intravenous immunoglobulin (25 g daily for 5 days) and methylprednisolone (250 mg daily for 5 days)				
2	First-line therapy	ns	At the 2-month follow-up, her mood state, appetite and sleep were satisfactory. No abnormalities in cognition or behavior were found	Improvement	Rong et al. [7]
	Intravenous immunoglobulin (20 g daily for 5 days) and methylprednisolone (1000 mg daily for 5 days)				
3	ns	ns	ns	Lost to follow-up	Torgovnick et al. [8]
4	First-line therapy	ns	ns	Improvement	Caglayan et al. [9]
	Intravenous immunoglobulin (0.4 g/kg/day for 5 days) and methylprednisolone (500 mg daily for 5 days)				
	Second-line therapy				
	Rituximab (100 mg/week)				
5	First-line therapy	ns	At the 12-month follow-up, a significant recovery was obvious: she was able to drive and care for her kids. No further immunotherapy was required	Improvement	Simabukuro et al. [10]
	Plasmapheresis (6 sessions was conducted on alternating days)				
6	First-line therapy	ns	Her mental status and speech function improved and she was able to walk with assistance	Improvement	Hanagasi et al. [11]
	Intravenous immunoglobulin (1 g/kg for 5 days) and methylprednisolone (0.4 g/kg)				
7	No use	ns	At the 14-month follow-up, complete recovery of patient's catatonia with the presence of suspiciousness and mild mood swing	Improvement	Yoshimura et al. [12]
8	No use	ns	The patient's symptoms gradually resolved	Improvement	Yoshimura et al [12]
9	First-line therapy	The patient was discharged on prednisone 60 mg daily and was tapered off over the course of a year	At the 8-month follow-up, the patient's cognition returned to pre-morbid levels. At the 1-year follow-up, the patient's cognition was normal and there was no psychiatric or neurological return	Improvement	Heekin et al. [13]
	Intravenous immunoglobulin (0.4 g/kg/day for 5 days) and methylprednisolone (1000 mg daily for 5 days and a second course with 1000 mg daily for 5 days)				
10	First-line therapy	Two 5-day courses of intravenous immunoglobulin (25 g/day) were given 3 weeks and 3 months following the initial treatment course	At the 10-month follow-up, the patient recovered well and performed well on self-care and neuropsychological tests	Improvement	Huang et al. [14]
	Intravenous immunoglobulin (22.5 g/day for 5 days)				
11	First-line therapy	ns	Significant improvement in psychiatric symptoms, social functioning, emotional reactions, and memory functioning	Improvement	Conroy et al. [15]
	Intravenous immunoglobulin (ns)				
	Second-line therapy				
	Cyclophosphamide (750 mg per square meter, each time) for a total of two times				
	Rituximab (375 mg per square meter, each time) for a total of four times				
12	First-line therapy	ns	At the three year follow-up, the patient's neurological examination was normal, with significant improvement in neuropsychological assessment	Improvement	Ponte et al. [16]
	Methylprednisolone (1000 mg daily for 5 days) and Intravenous immunoglobulin (0.4 g/kg/d for 5 days)				
	Prednisolone (60 mg/day for 20 days, then switched to 50 mg/day for 36 days)				
	Second-line therapy				
	Cyclophosphamide (monthly treatments)				
13	First-line therapy	The patient received her fourth dose of rituximab in the week following her discharge and has recovered fully since then	The patient received close neuropsychiatric follow-up and remained in a sound mood, sleeping well, without any signs of panic attacks or perceptual disturbances	Improvement	Fields et al. [17]
	Intravenous immunoglobulin (5 days)				
	Second-line therapy				
	Rituximab (once a week for three times)				
14	No use	ns	The patient's aggression improved with no obvious subsequent complications	Improvement	Kurita et al. [18]

ns, no statement

### Treatment and prognosis

**Fig. 3:**
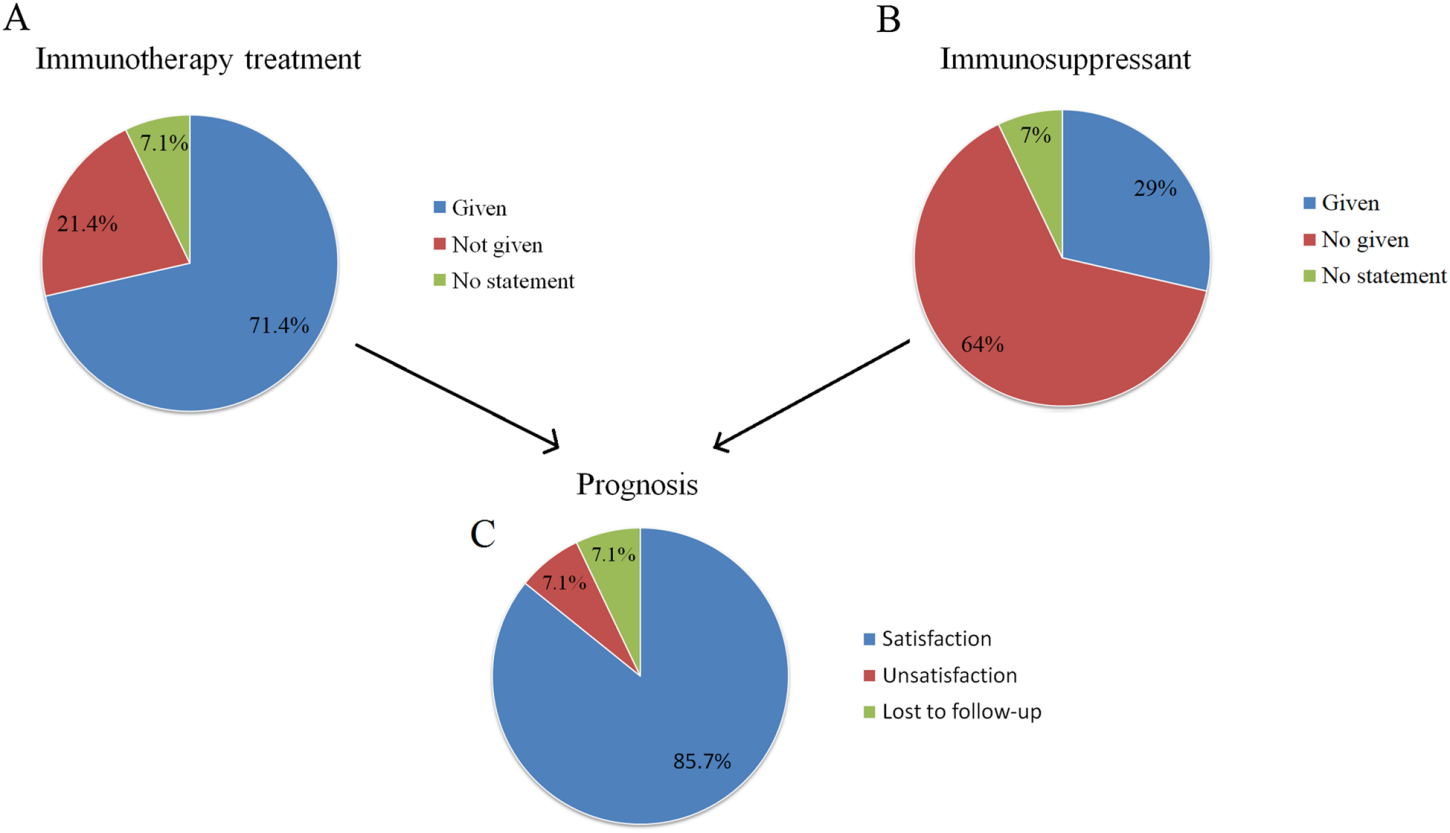
Treatment and prognosis of anti-NMDA receptor encephalitis patients with a long-term history of mental disorders. **A** Immunotherapy usage, **B** second-line immunotherapy (immunosuppressant) usage and **C** prognosis

Ten patients (71.4%) received immunotherapy (first-line and second-line), including intravenous immunoglobulin, corticosteroids, plasmapheresis, cyclophosphamide, and rituximab. Three of them (21.4%) did not receive any immunotherapy and one patient’s therapeutic information was not very clear (Fig. 3A). Of all patients, four patients (29%) received immunosuppressant, including cyclophosphamide and rituximab (Fig. 3B). Clinical symptoms improved significantly after treatment in 12 patients (85.7%). One patient died and one patient was lost to follow-up (Fig. 3C).

## Discussion

It is well accepted that acute psychiatric disturbance is one of the important clinical characteristics of anti-NMDA receptor encephalitis [1, 2, 5]; however, the features of anti-NMDA receptor encephalitis patients with a long-term history of mental disorders remain unclear. Identification of these clinical features may help clinicians obtain a broader and better understanding of anti-NMDA receptor encephalitis. It is also important to improve early accurate diagnosis and satisfactory prognosis because many patients could be misdiagnosed as other disorders due to overlapping psychiatric symptoms.

In this study, we found that patients with newly diagnosed anti-NMDA receptor encephalitis combined with a long history of mental disorders ranged in age from 16 to 52 years, with a mean of 35 years, the majority were adult females, and tended to be seen first in psychiatry. These long-term mental disorders included depressive disorders, bipolar disorder, schizoaffective disorder, schizophrenia, attention deficit hyperactivity disorder, and autism spectrum disorder and range from 1 to 22 years, with 9.8 years on average.

Clinical features of newly diagnosed anti-NMDA receptor encephalitis patients with a long-term history of mental disorders include speech impairment (71.4%); abnormal behaviors (64.3%); catatonia (64.3%); rigidity and hallucinations (42.9%); fever, seizures, and insomnia (35.7%); and confusion, agitation, delusion, memory deficits, and dyskinesia (28.6%). By contrast, common presentations of patients with anti-NMDA receptor encephalitis without a previous psychiatric medical history are seizures, dyskinesia, disorientation/confusion, and mutism/staring [1, 19, 20]. Our results indicate that clinical features of mental and behavioral impairments are more frequently observed in newly diagnosed anti-NMDA receptor encephalitis patients with a long-term history of mental disorders than those without mental illness. Most patients could be misdiagnosed as having other mental illnesses by psychiatrists, despite the presence of neurological comorbidities [11]. Furthermore, no remarkable neuroimaging findings were noted in these patients. This finding can partly explain why many patients first visit a psychiatry rather than a neurology department [11].

Despite many variabilities of clinical features and the lack of well-established pathognomonic symptoms, certain evidence may help clinicians to differentiate anti-NMDA receptor encephalitis from previous psychiatric disorders. Unlike gender patterns of primary mental illness [21, 22], most reported cases of newly diagnosed anti-NMDA receptor encephalitis patients with a long-term history of mental disorders were adult females. In our study, anti-NMDA receptor encephalitis with a long-term mental disorder was more prevalent in women (with a female-to-male ratio of 11:3). Women have a higher lifetime prevalence of psychiatric disorders than men [23]. For example, the ratio of women to men with major depression is 1.7:1 [24]. However, the prevalence of anti-NMDA receptor encephalitis is higher in women than the prevalence of psychiatric disorders alone, with a female-to-male ratio of 8:2 [1], which is similar to our findings. Furthermore, psychiatric symptoms subsequently followed by neurological comorbidities, including seizures, dyskinesia, and rapid memory deficits, were frequently noted in anti-NMDA receptor encephalitis with coexisting mental disorders, which was not evident in primary psychiatric disorders [25]. Moreover, most patients included in our study achieved a good prognosis after immunotherapy, while only antipsychotics are effective for primary psychiatric disorders [26]. We propose that in the case of individuals with long-term psychiatric disorders, sudden fluctuations of psychiatric symptoms may serve as a potential warning signal for clinicians to suspect a possible diagnosis of anti-NMDA receptor encephalitis.

Previous research has reported that anti-NMDA receptor encephalitis antibodies have been detected in the serum of 2/70 patients with major depressive disorders [27]. It is of interest to explore the relationship between the presence of such positive serum antibodies in psychiatric patients [27] and anti-NMDA receptor encephalitis with long-term psychiatric patients in our study. We believe the findings of above study may not be specifically associated with patients presenting with anti-NMDA receptor encephalitis in long-term psychosis, for the following reasons. First, the repertoire of anti-NMDA receptor antibody subtypes in the study of 70 patients is different from the specific anti-NR1a IgG autoantibodies that directly caused anti-NMDA receptor encephalitis [28]. Steiner and colleagues demonstrated that 7/121 patients with schizophrenia were positive for anti-NMDA receptor IgA and/or IgM autoantibodies, but not IgG autoantibodies against NR1a (except NR1a/NR2b) [27]. Second, detection of anti-NMDA receptor encephalitis antibodies in CSF is more meaningful for the diagnosis of anti-NMDA receptor encephalitis than in serum [1, 2]. In our study, 93% of patients showed positive anti-NMDA receptor antibodies in the CSF, which is significantly different from the study in which positive antibodies were present in serum [27]. Future studies need to further explore the mechanisms underlying the occurrence of NDMA receptor antibody positivity in psychiatric patients without signs of encephalitis and the relationship of this condition to anti-NMDA receptor encephalitis.

Immunotherapy, including intravenous immunoglobulin, corticosteroids, plasmapheresis, or immunosuppressant (cyclophosphamide and rituximab), is the main therapeutic option. The majority of the patients in our study achieved satisfactory outcomes after the above therapy, which is consistent with previous studies in pure anti-NMDA receptor encephalitis [2, 28].

## Conclusion

In summary, anti-NMDA receptor encephalitis patients with a long-term history of mental disorders is a relatively rare and underestimated condition, particularly when patients did not first visit the neurology department. Being an adult female may be a risk factor for developing anti-NMDA receptor encephalitis coexisting with long-term mental disorders. The most common clinical signs of these patients are speech impairment, abnormal behaviors, and catatonia, which are more common features, compared to the clinical feature of patients with anti-NMDA receptor encephalitis without a psychiatric history. Immunotherapy is the main therapeutic option and the patients’ prognosis can be good after treatment. Clinically, it may serve as a significant clue to suspect a diagnosis of anti-NMDA receptor encephalitis when a patient with long-term psychiatric disorders shows new-onset acute neuropsychiatric symptoms or exacerbated previous psychiatric symptoms.

## Data Availability

All data generated or analyzed during this study are included in this published article.

## Abbreviations

NMDA: Anti-*N*-methyl-d-aspartate
CSF: Cerebrospinal fluid
MRI: Magnetic resonance imaging
